# Development of a Hamster Natural Transmission Model of SARS-CoV-2 Infection

**DOI:** 10.3390/v13112251

**Published:** 2021-11-09

**Authors:** Stuart Dowall, Francisco J. Salguero, Nathan Wiblin, Susan Fotheringham, Graham Hatch, Simon Parks, Kathryn Gowan, Debbie Harris, Oliver Carnell, Rachel Fell, Robert Watson, Victoria Graham, Karen Gooch, Yper Hall, Simon Mizen, Roger Hewson

**Affiliations:** 1 United Kingdom Health Security Agency (UKHSA), Porton Down, Salisbury SP4 0JG, UK; Javier.Salguero@phe.gov.uk (F.J.S.); nathan.wiblin@phe.gov.uk (N.W.); susan.fotheringham@phe.gov.uk (S.F.); graham.hatch@phe.gov.uk (G.H.); simon.parks@phe.gov.uk (S.P.); kathryn.gowan@phe.gov.uk (K.G.); debbie.harris@phe.gov.uk (D.H.); oliver.carnell@phe.gov.uk (O.C.); rachel.fell@phe.gov.uk (R.F.); robert.watson@phe.gov.uk (R.W.); Victoria.Graham@phe.gov.uk (V.G.); karen.gooch@phe.gov.uk (K.G.); Yper.Hall@phe.gov.uk (Y.H.); roger.hewson@phe.gov.uk (R.H.); 2 Tecniplast UK Ltd., BCM Box 3058, London WC1N 3XX, UK; simon@tecniplastuk.com

**Keywords:** COVID-19, transmission, animals

## Abstract

The global pandemic of coronavirus disease (COVID-19) caused by infection with severe acute respiratory syndrome coronavirus-2 (SARS-CoV-2) has led to an international thrust to study pathogenesis and evaluate interventions. Experimental infection of hamsters and the resulting respiratory disease is one of the preferred animal models since clinical signs of disease and virus shedding are similar to more severe cases of human COVID-19. The main route of challenge has been direct inoculation of the virus via the intranasal route. To resemble the natural infection, we designed a bespoke natural transmission cage system to assess whether recipient animals housed in physically separate adjacent cages could become infected from a challenged donor animal in a central cage, with equal airflow across the two side cages. To optimise viral shedding in the donor animals, a low and moderate challenge dose were compared after direct intranasal challenge, but similar viral shedding responses were observed and no discernible difference in kinetics. The results from our natural transmission set-up demonstrate that most recipient hamsters are infected within the system developed, with variation in the kinetics and levels of disease between individual animals. Common clinical outputs used for the assessment in directly-challenged hamsters, such as weight loss, are less obvious in hamsters who become infected from naturally acquiring the infection. The results demonstrate the utility of a natural transmission model for further work on assessing the differences between virus strains and evaluating interventions using a challenge system which more closely resembles human infection.

## 1. Introduction

The outbreak of coronavirus disease (COVID-19), caused by the etiological agent severe acute respiratory syndrome coronavirus-2 (SARS-CoV-2), was declared a pandemic on 11 March 2020 [1]. It is continuing to have a global impact on public health, and the development of new countermeasures to mitigate the infection continues apace.

For the testing of vaccines and antivirals, and studying the disease pathogenesis, there is currently no substitute for animal models. The hamster model is now widely utilised as a small animal model, in part due to having high similarities to the human ACE2 used for SARS-CoV-2 cellular entry with only four differences in 29 amino acids in the virus-receptor contacting region [2]. After challenge, hamsters exhibit a consistent lung disease phenotype and immune responses consistent to those observed in humans with pneumonia [3].

Data from epidemiological studies provide evidence that the principal mode of SARS-CoV-2 infection is via airborne transmission [4], with infectious SARS-CoV-2 particles being recovered from air samples collected from a hospital ward housing COVID-19 patients [5]. Therefore, to model this challenge route in vivo, we sought to develop a COVID-19 natural transmission exposure for hamsters to resemble this route as an alternative to the widely used intranasal challenge.

## 2. Materials and Methods

### 2.1. Animals

Golden Syrian hamsters aged 6–8 weeks were obtained from a UK Home Office accredited facility (Envigo RMS UK Ltd., Bicester, UK). The animals were housed in cages that are designed in accordance with the requirements of the UK Home Office Code of Practice for the Housing and Care of Animal Used on Scientific Procedures (1986). During procedures with SARS-CoV-2, the animals were housed in a flexible-film isolator within a Containment Level 3 facility. The animals were randomly put into groups and individually housed, with equal allocation of male and female animals to each study. For direct intranasal challenge studies, groups sizes of 6 hamsters were used as the minimal number required for statistical significance to be achieved. For natural transmission studies, 8 donor and 16 recipient animals were used. This configuration enabled the use of a single flexible-film isolator and provided data for the justification of future group sizes using this system. Access to food and water was ad libitum and environment enrichment was provided. Rooms were maintained within set parameters: 20–24 °C, 45–65% humidity and a 12/12 light cycle.

All experimental work was conducted under the authority of a UK Home Office approved project licence that had been subject to local ethical review at Public Health England (now part of the UK Health Security Agency (UKHSA)) Porton Down by the Animal Welfare and Ethical Review Body (AWERB) as required by the Home Office Animals (Scientific Procedures) Act 1986.

### 2.2. Virus Challenge

SARS-CoV-2 Victoria/01/2020 [6] was generously provided by the Peter Doherty Institute for Infection and Immunity, Melbourne, Australia at P1 and passaged twice in Vero/hSLAM cells (Cat#04091501) obtained from the European Collection of Cell Cultures (ECACC), UK. Virus titre was determined by plaque assay on VeroE6 cells (ECACC Cat#85020206). Challenge substance dilutions were made in sterile phosphate buffered saline (PBS) with delivery via intranasal instillation (200 µL total with 100 µL per nare) with animals being sedated using isoflurane.

### 2.3. Dose Response Experimental Design

As the shedding of virus would be critical for natural transmission studies, a study was designed to compare 2 challenge doses. Groups of 6 hamsters were intranasally challenged with a low dose (16 pfu) or a moderate dose (27,000 pfu) of SARS-CoV-2 and assessed for 7 days post-challenge. To reduce the number of animals used in experimental studies, mock-challenged hamsters were not included.

### 2.4. Natural Transmission Experimental Design

Designs for natural transmission infection were conducted through discussions with PHE scientists covering expertise areas in biosafety, in vivo experimental infections and virology along with representatives from Tecniplast, UK. The final design consisted of 3 plastic rodent cages (1500U, Tecniplast, London, UK) with individually-ventilated cage (IVC)-type plastic lids linked through their sidewalls with 85 mm plastic tubing to provide a flow of air from the central cage to the outer cages and ensure the animals could not reach each other. Between the plastic tubing was a 3.5 mm × 3.5 mm grid to prevent the transfer of large particles. The central lid had a 90 mm diameter hole with a pre-filter enabling air to come through the central cage. The outer walls of the 2 outer cages were fitted with small fans to support the airflow from the central cage to the 2 adjacent cages. To measure the airflow across the cages, a mass flow meter was used (TSI instruments model 4040).

To evaluate natural transmission infection, 8 donor animals were intranasally challenged with 1.1 × 10^4^ pfu SARS-CoV-2. These were housed in the central cage, with naïve recipient hamsters housed in the 2 adjacent cages. To ensure an equal gender distribution, the left cage contained male animals whereas the right cage contained female animals. The study lasted for 14 days.

### 2.5. Clinical Observations

The animals were monitored for clinical signs of disease by experienced husbandry and animal welfare staff twice daily for the entirety of the studies. Clinical signs of disease were assigned a score based upon the following criteria: 0, normal; 1, behavioural changes; 2, ruffled fur, dehydrated, wet tail; 3, arched, wasp-waisted, eyes shut; and 5, laboured breathing. A cumulative score to combine all of the observed signs was then assigned for each animal at that timepoint.

At the same time each day, the animals were weighed throughout the studies.

### 2.6. Sampling

Throat swabs were taken throughout the studies. A dry flocked mini-tip swab (product MW002NF, MWE, Corsham, UK) was used for sampling before adding to 1ml Virocult universal transport medium (product MW951T, MWE, Corsham, UK). For RNA extraction, these were inactivated in an AVL buffer (Qiagen, Manchester, UK) and ethanol. Downstream RNA extraction was performed using the BioSprint one-for-all vet kit (Indical Bioscience, Leipzig, Germany) and Kingfisher Flex platform (Thermo-Fisher, Loughborough, UK) as per the manufacturer’s instructions.

At the end of the studies, the animals were anaesthetized with isoflurane followed by a lethal dose of sodium pentobarbitone. During necropsy, a thoracic pluck and the whole head was sampled into 10% neutral-buffered formalin for histological analysis of the lung and nasal cavities.

### 2.7. Quantification of Viral Loads by RT-qPCR

A reverse transcription-quantitative polymerase chain reaction (RT-qPCR) targeting a region of the SARS-CoV-2 nucleocapsid (N) gene was used to determine viral loads and was performed using TaqPath™ 1-Step RT-qPCR Master Mix, CG (Applied Biosystems™), 2019-nCoV CDC RUO Kit (Integrated DNA Technologies, Leuven, Belgium) and QuantStudio™ 7 Flex Real-Time PCR System. Sequences of the N1 primers and probe were: 2019-nCoV_N1-forward, 5′ GACCCCAAAATCAGCGAAAT 3′; 2019-nCoV_N1-reverse, 5′ TCTGGTTACTGCCAGTTGAATCTG 3′; 2019-nCoV_N1-probe, 5′ FAM-ACCCCGCATTACGTTTGGTGGACC-BHQ1 3′. The cycling conditions were: 25 °C for 2 min, 50 °C for 15 min, 95 °C for 2 min, followed by 45 cycles of 95 °C for 3 s, and 55 °C for 30 s. Samples were run in duplicate. The quantification standard was in vitro transcribed RNA of the SARS-CoV-2 N ORF (accession number NC_045512.2) with quantification between 1 × 10^1^ and 1 × 10^6^ copies/µL.

### 2.8. Foci-Forming Unit (FFU) Assay

Throat swab samples were serially diluted before being added, in duplicate, to a Vero E6 cell monolayer in a 96-well flat bottomed culture plate (seeded 24 h before) for 1 h at 37 °C. Samples were removed and an overlay media added, then incubated for 24 h at 37 °C. Plates were fixed overnight by adding 20% formalin and then fumigated. The following day the plates were washed with water and incubated with 0.3% hydrogen peroxide for 20 min at room temperature. The plates were washed again with sterile PBS and incubated with a rabbit anti-SARS-CoV-2 primary antibody (Cat no. 40592-T62, Sino Biologicals, Eschborn, Germany) for 1 h at room temperature, washed and incubated a further hour with a secondary goat anti-rabbit IgG HRP conjugate (Cat no. G21234, Invitrogen, Paisley, UK). After a further PBS wash, the plates were incubated for 10 min with TrueBlueTM substrate, washed with water, and left to dry for a minimum of 3 h, before counting using the CTL scanner.

### 2.9. Histopathological Analysis

Samples from the lungs, together with the nasal cavity, were fixed by immersion in a 10% neutral-buffered formalin and processed routinely into paraffin wax. Nasal cavity samples were decalcified using an EDTA-based solution prior to embedding. Sections were cut to 4 µm and stained with haematoxylin and eosin (H&E) and examined microscopically. In addition, samples were stained using the RNAscope technique to localise SARS-CoV-2 virus RNA. Briefly, tissues were pre-treated with hydrogen peroxide for 10 min (room temperature), target retrieval for 15 min (98–101 °C) and protease plus for 30 min (40 °C) (Advanced Cell Diagnostics, Abingdon, UK). A V-nCoV2019-S probe (Cat No. 848561, Advanced Cell Diagnostics) was incubated on the tissues for 2 h at 40 °C. Amplification of the signal was carried out following the RNAscope protocol using the RNAscope 2.5 HD Detection kit—Red (Advanced Cell Diagnostics). The slides were scanned digitally using a Hamamatsu S360 digital slide scanner and examined using ndp.view2 software (v2.8.24). Nikon NIS-Ar software was used to perform digital image analysis and qualified veterinary pathologists selected the areas of SARS-CoV-2 induced bronchopneumonia using digitalised slides and the image analysis software in order to calculate the area of pneumonia and quantify the presence of viral RNA in the lung sections. Digital image analysis was carried out and the total area of the lung section which was positive for viral RNA was calculated.

A semiquantitative scoring system was applied to evaluate the severity of lesions in the lung and nasal cavity (Table S1). For the nasal cavity, a semiquantitative scoring system was applied to evaluate the presence of virus RNA: 0=no positive staining; 1 = minimal; 2 = mild; 3 = moderate and 4 = abundant staining.

### 2.10. Statistical Analysis

Statistical analyses were performed using MiniTab, v.16.2.2 (Minitab Inc, State College, PA, USA). A non-parametric Mann-Whitney statistical test was applied to ascertain significance between groups. A significance level below 0.05 was considered statistically significant.

## 3. Results

### 3.1. Unique Cage Design for Modelling Natural Transmission

**Figure 1:**
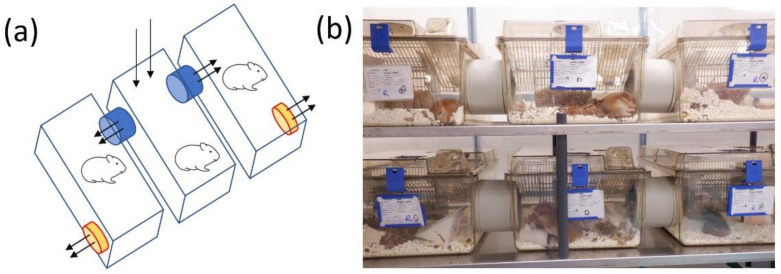
Design of natural transmission modelling cages. (**a**) Diagrammatic overview with arrows denoting air movements from central cage and across into side cages. (**b**) Photo of cages set up inside the flexible-film isolator housing hamsters.

In order to design a suitable cage system, prototypes were tested for practicalities of handling within the flexible-film isolators used for housing animals challenged with SARS-CoV-2. The final design comprised a set of 3 cages, each linked through a 130 mm diameter connector and with small fans on the outsides of the side cages to draw air from the central cage to the adjacent cages (Figure 1a). Fans were drawing 12.5 L/min air, equating to 34 air changes per hour (ACH) for the central cage and 17 ACH for each of the side cages. Within the flexible-film isolator, the cages were placed on shelving units allowing full access to animals for observations, handling and husbandry (Figure 1b).

### 3.2. Viral Shedding Is Similar between Hamsters Challenged with Two Different Doses of Virus

**Figure 2:**
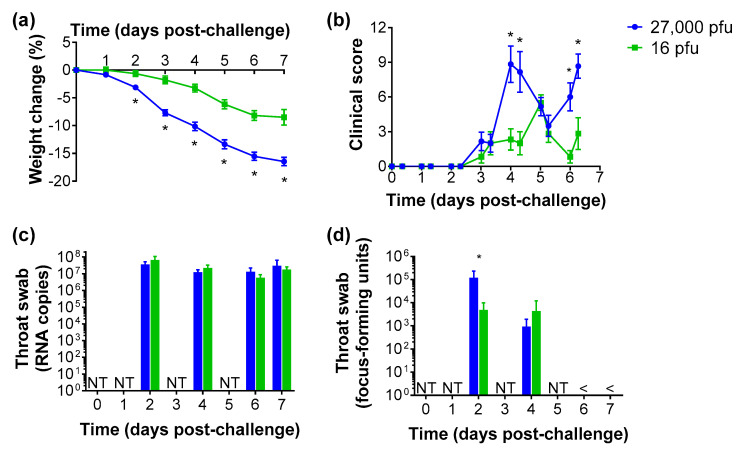
Clinical and virological findings in hamsters intranasally challenged with 16 and 27,000 pfu SARS-CoV-2. (**a**) Weight loss compared to the day of challenge. (**b**) Clinical score. (**c**) Viral RNA levels in throat swabs. (**d**) Quantification of live virus in throat swabs. Data show mean values with error bars denoting standard error from *n* = 6 hamsters per group. *, *p* < 0.05 (Mann-Whitney test); NT, not tested as samples not collected at these timepoints; and <, below the assay detection limits.

**Figure 3:**
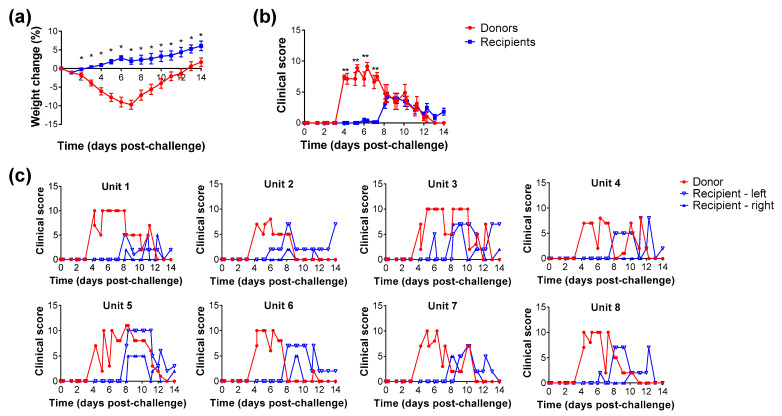
Clinical observations in donor hamsters challenged with SARS-CoV-2 and recipient animals housed adjacently. (**a**) Weight change shown as percentage compared to the day of challenge. (**b**) Average clinical scores for donor and recipient groups. Lines indicate mean value with error bars denoting standard error (*n* = 8 donor animals and *n* = 16 recipient animals); *, *p* < 0.05 (Mann-Whitney test). (**c**) Clinical scores from each of the 8 set-ups used, each containing one donor animal and a recipient hamster on either side. Lines show results from each individual animal.

Hamsters were intranasally challenged with a low (16 pfu) and moderate (27,000 pfu) dose of SARS-CoV-2 to compare the kinetics of virus loads. Weight loss was observed in all challenged hamsters (Figure 2a), with a significant difference between groups from day 2 onwards (*p* < 0.05, Mann-Whitney test). Similarly, clinical signs were observed from day 3 post-challenge (Figure 2b), with significantly increased scores on days 4 and 6 in those receiving the higher dose (*p* < 0.05, Mann-Whitney test). Throat swabs were collected on every second day post-challenge, with SARS-CoV-2 viral RNA being detected at all timepoints (Figure 3c). To determine the presence of live virus, throat swabs samples were assessed in a focus-forming unit (ffu) assay with virus detected on day 2 and day 4 post-challenge but undetectable from day 6 (Figure 3d). At day 2 this was significantly higher in the high challenge group (*p* < 0.05, Mann-Whitney test), but by day 4 the levels were comparable.

### 3.3. Natural Transmission of SARS-CoV-2 to Naïve Recipients

#### 3.3.1. Clinical Parameters

Donor hamsters were intranasally challenged and housed in the central cage, with a single recipient hamster housed in each of the adjacent cages. The weight loss in donor animals successively increased each day, reaching a peak at day 7 post-challenge, whereas in the recipient animals there was a pause in weight gain on days 6–8 but no significant loss was observed (Figure 3a). In the donor animals, clinical signs were first shown on day 4 post-challenge, whereas in the recipient animals these were delayed and were often at lower levels than in hamsters directly challenged (Figure 3b). Results from the individual units show consistency of the later induction of clinical signs developing and differences in the individual recipient animals (Figure 3c).

#### 3.3.2. Viral Loads

**Figure 4:**
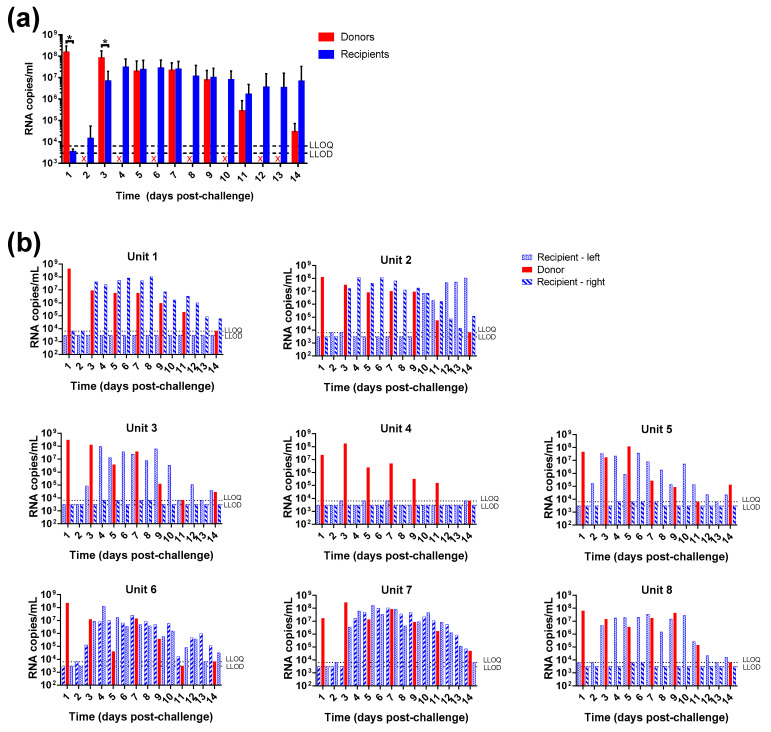
Viral RNA detection in throat swabs of donor and recipient hamsters. Recipient animals were sampled daily whereas donor animals were swabbed less frequently. (**a**) Average levels from donor and recipient groups. Bars show mean values with error bars denoting standard error. *, *p* < 0.05 (Mann-Whitney test); X denotes where samples were not scheduled from donor animals. (**b**) Results from individual units and animals. LLOQ, lower limit of quantification; LLOD, lower limit of detection.

Due to a high sensitivity and being the gold standard for diagnostic assays, RT-qPCR was used to detect the presence of virus [7]. SARS-CoV-2 viral RNA was detected in the throat swabs of all challenged donor animals throughout the course of the study and in 87.5% (14/16) of recipient animals (Figure 4). The concentration of viral RNA varied in recipient animals, with 62.5% (10/16) reaching levels similar to those seen in hamsters intranasally-challenged from 3 days since initial exposure to the donor animal.

#### 3.3.3. Histopathology

**Figure 5:**
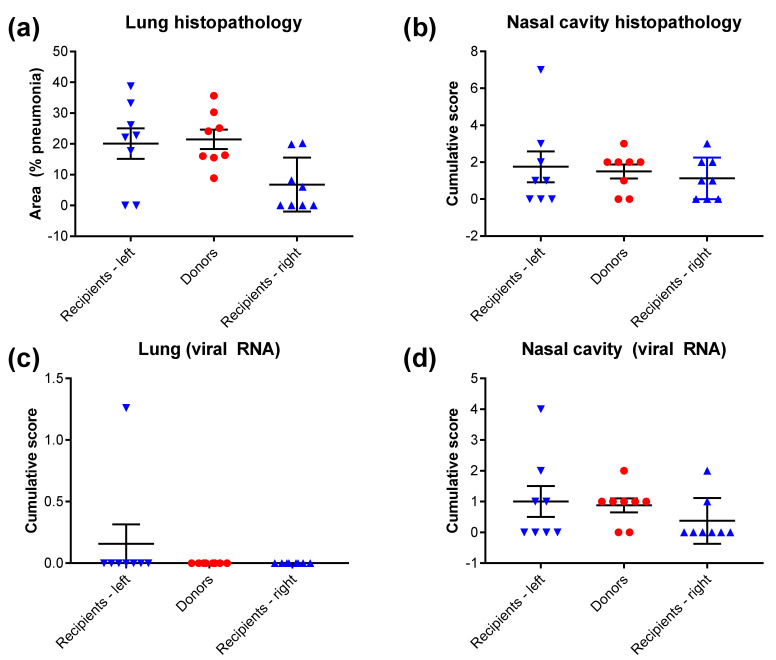
Histopathology results from recipient animals in left and right cages 14 days post-housing adjacent to donor animals. (**a**) Lung histopathology as percentage area affected by pneumonia. (**b**) Nasal cavity histopathology as cumulative score from exudate and necrosis. (**c**) Viral RNA levels in the lung. (**d**) Viral RNA levels in the nasal cavity. Results from individual animals shown (*n* = 8 per group) with mid-line showing mean value and error bars denoting standard error. No statistically significant findings were observed between left and right recipient groups (*p* > 0.05, Mann-Whitney test).

**Figure 6:**
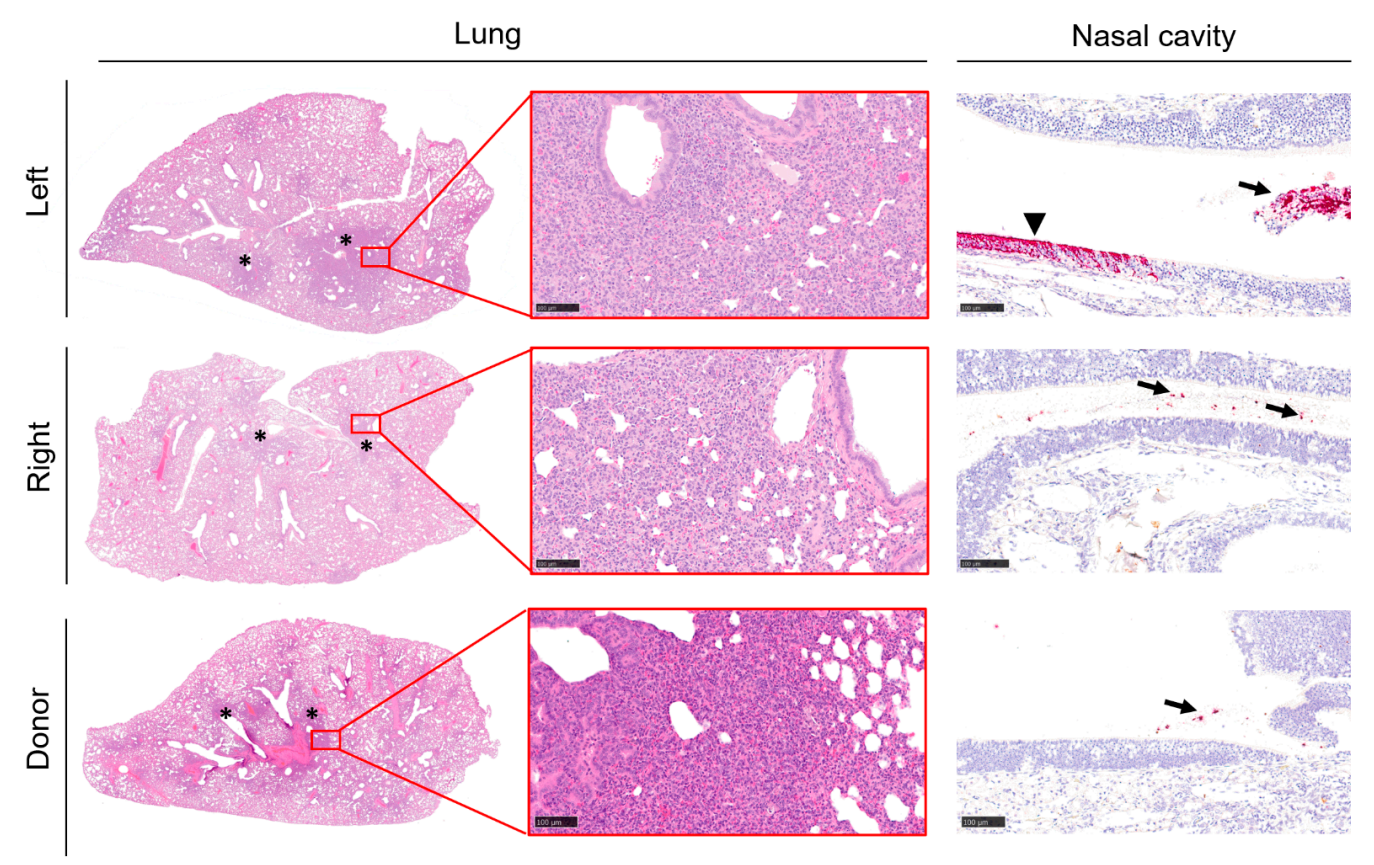
Histopathology of the lung and presence of SARS-CoV-2 RNA (RNAScope ISH) in the nasal cavity. Areas of pneumonia (*) were observed in animals housed on both the left and the right side of the donor animals. Virus RNA was detected in the nasal cavity, mostly within the nasal exudates (arrows), but also in a few epithelial cells (arrowhead). Bar = 100 µm.

The lung and nasal cavity from the recipient animals were analysed at the end of the study (14 days post-housing adjacent to donor animals) for histological changes and the presence of viral RNA via in situ hybridisation. Results demonstrated changes in most recipient animals, with pneumonia detected in the lungs and histopathological change in 62.5% (10/16) of hamsters (Figure 5a,b). Viral RNA was only detected in the lung from a single recipient animal and in the nasal cavity of 37.5% (6/16) of recipient animals (Figure 5c,d). There were no significant differences (*p* > 0.05, Mann-Whitney test) in the histopathology findings between recipient animals housed on the left or right side of the donor animal. Representative images show observations in the tissues (Figure 6), with differences between donor and recipient animals attributed to the state of infection at the time of post-mortem, as the recipient animals were infected at a later timepoint.

## 4. Discussion

This study describes a capability to study SARS-CoV-2 in a natural transmission setting which resembles infection through airborne exposure. A preliminary intranasal infection study was conducted to compare two challenge doses in order to ascertain whether a lower inoculum dose resulted in longer kinetics of shedding, as observed in the influenza ferret model [8]. However, despite a milder disease observed in low dose (16 pfu) challenged hamsters compared to those receiving a moderate (27,000 pfu) dose, illustrated by significantly less weight loss and lower clinical scores, the levels of virus shedding for low and moderate doses were similar. The intranasal challenge dose for hamsters is often reported in the region of 10^5^ virus particles [2,9,10,11]. Viral RNA was detected throughout the course of the study, with no difference between challenge doses, and live virus was detected up to day 4 before being undetectable at day 6 in both groups. To ensure a consistent and reliable infection, the moderate challenge dose was used for future experiments for infecting the donor animals in the natural transmission model.

When naïve hamsters were housed in adjacent cages to the challenged animals, clinical signs of disease were observed in all recipient hamsters generally 4 days after clinical signs were detected in the donor animals. The manifestation of clinical scores varied, with some hamsters only having mild signs (e.g., ruffled fur) whereas others had clinical scores the same as the directly-challenged hamsters. Whilst weight loss is a key indicator of disease in intranasally-challenged hamsters, for those infected via natural transmission this was less of a reliable readout. The data which showed that not all recipient hamsters demonstrated disease manifestation is consistent with others where asymptomatic infection was detected in an airborne transmission model and the course of disease was distinctly different compared to direct fomite exposure [10]. The virus challenge dose used in the second study was lower than used for the moderate dose in the first study (11,000 pfu vs. 27,000 pfu, respectively). These doses were determined from back titration of the challenge virus preparation, so this variation was expected.

Viral RNA present in throat swabs was another indicator of infection in the recipient animals, with virus detectable in 87.5% of recipient animals. Thus, for countermeasure evaluation in the natural transmission model the size of groups will need adjusting to ensure statistical significance of the effect as not all recipient animals may become infected.

Histology data showed changes in the lung, but viral RNA was only detected in a single recipient animal. In contrast, histological changes and viral RNA were detected in the nasal cavity of most recipient animals, suggesting that the infection was focused in the upper respiratory tract at the time of death and had cleared from the lower respiratory tissues 14 days since living alongside infected animals. Previous studies have demonstrated that pathological changes in SARS-CoV-2 infected hamsters have resolved within 14 days [12].

The variation in RT-qPCR levels, clinical outcomes and histological findings were expected, given the variability in virus exposure including several compounding factors of the animals such as activity and their individual susceptibility (due to the outbred nature of the hamster species). Given the infectious dose for Syrian hamsters is low, with previous reports showing that approximately five infectious particles is enough to cause infection in 50 % of animals [13], our experimental system passes the threshold of infection for the majority of recipient animals. The variability of transmission is also influenced by other biological and physical factors, such as the concentration of inoculum, the viral integrity and the ability of virions to survive desiccation and aerosolisation, in addition to environmental factors (air movement, temperature and humidity) and host natural defences [14].

Transmission studies with SARS-CoV-2 in hamsters have previously been reported and can serve as useful tools to better understand infection. When the D614G variant was detected, the hamster model was used to demonstrate similarities in viral loads compared to wild-type virus, but interestingly a faster transmission phenotype [15]. Unfortunately, Hou et al. [15] did not clearly identify aerosol transmission and apart from the naïve hamster being adjacent to a cage with an infected animal, scant details were provided on the physical experimental set-up. For some direct contact studies on transmissibility, animals have been housed within the same cage [2,9,11,16]; however, given that fomite exposure results in SARS-CoV-2 infection [9,10] the exact route of transmission cannot be fully ascertained. To further separate animals, partitions between co-housed hamsters have been used to allow airflow [9,10]. The cage system we describe has an enhanced physical separation, allowing the cages to be individually utilised for the monitoring and care of the animals so reducing potential cross-contamination. Other cage partition formats are mainly based on housing within individually ventilated cages (IVC) housed on specific racking systems [9,10], which limits customisation and the distance between donor and recipient animals. The new cage formats described within this report are housed in flexible-film isolators without these restrictions.

The design of the cages within this study will be amenable to studies to evaluate the contribution of particles of different sizes (e.g., droplets vs. aerosols), as has been performed for other respiratory viruses such as influenza [17]. By changing the size of the filter units and distances between the cages different particle sizes can be measured and contribute to a more informative study. Particle sizes 5 µm or smaller have a propensity to deposit in the lower respiratory tract [18] whereas those sized 6–12 µm deposit in the upper airways [19]. A standard dogma associated with respiratory infections is that they are mainly associated with large droplet transmission and close contact; however, this has recently been challenged and recent evidence from several different pathogen types has shown that the spread and transmission by smaller aerosols are more common along with close contact where the highest concentrations occur [14].

Whilst the work reported within was conducted with Golden Syrian hamsters, other hamster species have also been reported to serve as small animal models in SARS-CoV-2 research, including cardiomyopathy J2N-k [20] and Roborovski strains [21]. The set-up described could thus be transferable for these models too.

Our results contribute to the refinement and development of animal models for COVID-19, which can be used to increase the understanding of the pathogenesis to infection [22], as well as to provide a system with more clinically relevant infection for countermeasure testing.

## Supplementary Materials

The following are available online at https://www.mdpi.com/article/10.3390/v13112251/s1, Table S1: Scoring criteria for the subjective assessment of microscopic changes in lung and nasal cavity of hamsters infected with SARS-CoV-2.
