# Supplementary material for: Development of a Hamster Natural Transmission Model of SARS-CoV-2 Infection
Source: Viruses. 2021 Nov 9;13(11):2251. doi: 10.3390/v13112251 (PMC8625437; doi:10.3390/v13112251)
Supplement: Supplementary file 1 [file viruses-13-02251-s001.zip › viruses-1437987-supplementary.pdf]

viruses

Supplementary Table S1. Scoring criteria for the subjective assessment of microscopic changes in lung and nasal cavity of hamsters infected with SARS-CoV-2.

Location	Lesion	Score 0 (normal)	Score 1 (minimal)	Score 2 (mild)	Score 3 (moderate)	Score 4 (marked)
Lung	Airway epithelial degeneration/necrosis and/or inflammatory cell infiltration with or without exudates	None	Occasional (1 or 2) airways affected; **up to 5% of slide affected**	Present in multiple airways; **up to 25% of airways affected**	Present in multiple airways; **between 26–50% of airways affected**	Present in multiple airways; **over 50% of airways affected**
	Peri-airway inflammatory infiltrates (cuffing)	None	Occasional incomplete, or loosely formed cuffs; **up to 5% of slide affected**	Numerous cuffs; predominantly incomplete and loosely formed with lesser well-formed complete cuffs; **up to 25% of airways affected**	Numerous cuffs; approximately half or more well-formed, and may have few broad, dense cuffs; between **26–50% of airways affected**	Numerous cuffs; predominantly well-formed with numerous broad, dense cuffs; **over 50% of airways affected**
	Perivascular inflammatory infiltrates (cuffing)	None	Occasional incomplete, or loosely formed cuffs; **up to 5% of slide affected**	Numerous cuffs; predominantly incomplete and loosely formed with lesser well-formed complete cuffs; **up to 25% of vessels affected**	Numerous cuffs; increased numbers are well-formed, and may have few broad, dense cuffs; **between 26–50% of vessels affected**	Numerous cuffs; predominantly well-formed with numerous broad, dense cuffs; **over 50% of vessels affected**
	Alveolar walls/space infiltration by inflammatory cells, primarily neutrophils and macrophages; variable oedema/fibrin +/- type II pneumocyte hyperplasia	None	Small numbers of foci affected within the parenchyma; **up to 5% of slide affected**	Increased frequency of foci; **between 6-25% of the slide affected**	Multifocal coalescing or larger patches of parenchyma; **between 26–50% of the slide affected**	Large areas of parenchyma; **over 50% of the slide affected**
Nasal cavity	Epithelial attenuation/ and necrosis in the mucosa	None	Occasional epithelial cell degeneration; **up to 5% of slide affected**	Multifocal epithelial cell degeneration/necrosis; **between 6–25% of the slide affected**	Multifocal, sometime coalescing areas of epithelial cell degeneration/; **between 26–50% of the slide affected**	Multifocally extensive to coalescing areas of epithelial cell degeneration/necrosis; **over 50% of the slide affected**
	Presence of exudates within the nasal cavity lumen	None	Minimal presence of inflammatory cells; **up to 5% of slide affected**	Mild exudation of degenerate epithelial and inflammatory cells; **between 6–25% of the slide affected**	Moderate exudation of degenerate epithelial and inflammatory cells; **between 26–50% of the slide affected**	Marked/severe exudation of degenerate epithelial and inflammatory cells; **over 50% of the slide affected**
